# Diagnostics and monitoring in Hymenoptera venom allergy: current and future perspectives

**DOI:** 10.3389/falgy.2026.1757776

**Published:** 2026-04-24

**Authors:** Weronika Wilińska, Aleksandra Starosz, Kamil Grubczak

**Affiliations:** 1 Department of Regenerative Medicine and Immune Regulation, Medical University of Bialystok, Białystok, Poland; 2 Department of Allergology and Internal Medicine, Medical University of Bialystok, Białystok, Poland

**Keywords:** Hymenoptera venom allergy, KIT mutations, multi-omic molecular profiles, osteopontin, PGD_2_ metabolites, regulatory t-cell signatures

## Abstract

Hymenoptera stings are a major trigger of IgE-mediated anaphylaxis, yet only a minority of exposed individuals develop systemic reactions, making accurate diagnosis essential but complex. This review synthesizes current evidence across clinical, serologic, cellular and molecular diagnostic modalities in Hymenoptera venom allergy. Clinical history and epidemiologic predictors—such as occupation, cumulative sting exposure, reaction latency, comorbidities and quality-of-life impairment, provide crucial context but lack sufficient predictive power when used alone. Skin prick and intradermal testing remain first-line tools due to high sensitivity, although interpretation is limited by interspecies cross-reactivity, extract variability and reduced reliability shortly after a sting. Serum IgE and component-resolved diagnostics improve species identification but are influenced by cross-reactive carbohydrate determinants and cannot reliably predict reaction severity or venom-immunotherapy outcomes. Basal and acute tryptase measurements contribute significantly to risk stratification and detection of clonal mast-cell disorders, though normal values do not exclude severe reactions. Functional assays, including basophil activation testing, histamine-release assays and emerging mast-cell activation platforms, provide dynamic confirmation of effector-cell reactivity in diagnostically challenging cases. Controlled sting challenge remains the reference method for confirming clinical reactivity or protection but is reserved for selected high-risk patients due to inherent procedural risks. Novel biomarkers such as osteopontin, KIT mutations, PGD_2_ metabolites, regulatory T-cell signatures and multi-omic molecular profiles offer promising avenues for future refinement. Overall, evidence supports a multimodal, individualized diagnostic strategy integrating clinical context with complementary laboratory and functional tests.

## Introduction

Hymenoptera stings are among the most frequent causes of IgE-mediated anaphylaxis and represent a major public health concern in adults and children. Most people are stung at least once, but only a minority develop systemic reactions, with incidence estimated at 0.3%–7.5% in adults and about 3% in children (1). Clinical manifestations range from large local reactions to life-threatening multisystem involvement. In a five-year Swiss cohort of 143 adults, two-thirds had only local swelling, whereas one-third developed systemic reactions, including rare grade IV events and one fatality (2). Pediatric series show comparable vulnerability, with bee and wasp stings contributing equally to systemic reactions and nearly one-third reaching grade IV, often impairing daily functioning and emotional well-being (3).

The clinical heterogeneity of sting reactions reflects the biochemical complexity of Hymenoptera venoms. Honeybee venom contains enzymatic allergens such as phospholipase A_2_ (Api m 1), hyaluronidase (Api m 2), acid phosphatase (Api m 3), dipeptidyl-peptidase IV (Api m 5), peptides such as melittin and newer proteins including Api m 10 and Api m 11 (4). Yellow jacket venom carries homologous allergens (Ves v 1, Ves v 2, Ves v 5, Ves v 3), explaining frequent cross-reactivity in extract-based IgE assays. Although Api m 1 is the dominant honeybee sensitizer, several relevant proteins (e.g., Api m 10) are under-represented in therapeutic extracts, which may limit diagnostic accuracy and VIT efficacy. Comprehensive characterization of allergen repertoires is therefore crucial for both diagnosis and treatment optimization.

Diagnostic strategies have evolved accordingly. Skin prick testing (SPT), intradermal testing and venom-specific IgE remain the backbone of evaluation but are limited by false negatives shortly after stings, CCD-driven false positives and difficulty distinguishing primary sensitization from cross-reactivity. These problems are common in practice: many high-risk groups such as beekeepers report systemic reactions yet have never undergone formal evaluation (5). Asymptomatic sensitization in the general population further complicates interpretation when tests are read without clinical context.

To address these limitations, modern diagnostics increasingly employ component-resolved IgE testing with recombinant, CCD-free allergens, improving discrimination between genuine double sensitization and CCD-mediated cross-reactivity (4). Functional assays such as the basophil activation test (BAT) and mast-cell activation test (MAT) add information by directly assessing effector-cell responses. They are particularly useful when history, skin tests and IgE profiles are discordant or when multiple sensitizations create complex patterns. Emerging biomarkers and molecular tools, discussed as novel perspectives in diagnostics, further broaden the landscape and suggest future refinements in individualized risk assessment.

### Clinical history and epidemiologic risk factors in Hymenoptera venom allergy

Clinical history and epidemiologic context remain central in Hymenoptera venom allergy, yet reliance on patient recollection alone leads to under-recognition of high-risk individuals and delayed management. In >1,000 German beekeepers, systemic reactions were common, but many never underwent evaluation or counseling (5); similarly, many patients at a Warsaw referral center reported multiple systemic reactions before specialist assessment, with post-anaphylaxis care deviating from guidelines (6). Epidemiologic surveys refine referrals by identifying predictors: in nearly 1,000 subjects, male sex, older age, large local reactions, short latency, and prior systemic reactions independently predicted systemic events, supporting a structured questionnaire (7). Wasps were more frequent than bees as culprits, with systemic reactions linked to male sex, age >40, and rural residence (8); shorter inter-sting intervals and climate favored severe reactions (9). Multicenter and hospital-based data show a spectrum from cutaneous symptoms to shock, with greater severity in older patients and those with cardiovascular disease, and emphasize distinguishing IgE-mediated anaphylaxis from toxic envenomation after multiple stings (10, 11). Emergency data suggest stings are overlooked triggers in heavy drinkers, where alcohol and comorbidity obscure recognition (2). Occupational cohorts highlight risk shaping by exposure; outdoor jobs (including beekeeping) associate with more severe reactions (12), and cumulative sting count may serve as a simple risk marker (13); tailored tools efficiently capture exposure and burden (14).

Prognostically, large local reactions are common but rarely precede anaphylaxis, whereas prior systemic reactions strongly predict recurrence (15). In patients with at least Mueller grade II reactions, adult age and cardiovascular disease associated with greater severity, but baseline features alone offered modest predictive power (16). VIT decisions should integrate reaction grade with QoL impairment (123); latency and detailed skin symptoms are essential to risk assessment (17). The interplay between history and laboratory findings is critical: positive venom-sIgE without a convincing sting history is difficult to interpret, especially with multiple potential triggers (18); venom-sIgE does not reliably distinguish local from systemic reactors (19), and serology alone should not prompt VIT without supportive history (20). QoL studies reveal burdens under-represented in standard histories: adults with severe reactions or not yet on VIT report greatest impairment (21); children experience activity restriction and fear (22, 23); parental anxiety and lifestyle changes are substantial after severe pediatric reactions (24); validated QoL tools correlate with fear and functional impairment (25).

Systematic reviews translate these findings into practice: routine diagnostic work-up is not indicated after isolated large local or purely toxic reactions; detailed history should guide which patients with systemic reactions or high-risk exposures undergo testing and VIT, avoiding under- and overtreatment (26). Overall, clinical factors—including male sex, older age, outdoor work, rural residence, cumulative sting number, short latency, cardiovascular comorbidities, prior systemic reactions, and QoL impairment—refine risk assessment but cannot reliably predict future severity alone. They must be integrated with targeted diagnostic tests and, when indicated, venom immunotherapy to support individualized, evidence-based management.

## Methods

This narrative review used systematic methods to identify and synthesize clinical and diagnostic studies on Hymenoptera venom allergy. We searched PubMed, Embase, Web of Science and Cochrane Library for articles published between January 1970 and November 2025, supplemented by reference-list screening of selected papers and guidelines; search terms combined Hymenoptera/venom allergy, diagnostics (SPT/IDT, sIgE, CRD, CAP-inhibition), functional tests (BAT, MAT), biomarkers, sting challenge, VIT and quality of life. We included studies of adults and children with suspected or confirmed venom allergy, occupational/high-exposure cohorts, and patients with mast cell disorders; eligible designs were diagnostic accuracy studies, cohort studies, case-control studies, systematic reviews and guideline documents.

### Skin testing with Hymenoptera venom extracts: performance and pitfalls

Skin testing with standardized venom extracts remains a core diagnostic method in Hymenoptera venom allergy. Skin prick test (SPT) is performed with venom concentrations from 1 to100 µg/mL, and sometimes 300 µg/mL. If SPT is negative, intradermal test (IDT) should be performed using venom concentrations from 0.001 to 1.0 µg/mL, while in children IDT typically starts at 0.1 µg/mL because of lower skin sensitivity (27–29). Sequential IDT with incremental concentrations at ∼15-min intervals should be considered in patients with high risk of developing severe systemic reactions (e.g., mastocytosis) (29). Testing in those cases should be carried out in inpatient wards. However, patients with mastocytosis are at higher risk of false-negative skin tests despite prior systemic reactions to venom and therefore may require repeat skin or sIgE testing or alternative diagnostic methods (30).

Despite its standardization, multiple studies highlight important limitations of those tests. Early observations showed fluctuating hypersensitivity, with some patients developing renewed or intensified skin reactivity after systemic reactions. Wolf et al., reported that these changes did not parallel circulating IgE or IgG, suggesting a significant cellular component and demonstrating that skin reactivity cannot be interpreted as a simple surrogate of humoral sensitization (31). Long-term data confirm this complexity: in a cohort followed up to 16 years after VIT discontinuation, Golden et al., documented an overall decline in skin test positivity but persistent sensitivity in some patients. Positive tests were more common in those with multiple systemic reactions, collapse, aspirin use or asthma, yet skin test positivity did not reliably predict systemic reactions on re-sting (32, 33).

Comparisons with serologic testing further illustrate limitations of extract-based diagnostics. Ridolo et al., found that prick and intradermal tests confirmed sensitization in most symptomatic patients but rarely in asymptomatic individuals, although cross-reactivity, incomplete histories and heterogeneous exposure patterns complicated interpretation when skin and serology disagreed. Combining both methods reduced misclassification better than either alone (34). Karamloo et al., showed that variability in IgE-binding capacity among allergens within venom extracts can produce discrepancies between skin reactivity and serology, underscoring the importance of standardizing extract composition and recognizing biological differences between *in vivo* and *in vitro* assessments (35).

The evolution of skin test reactivity during and after VIT has been studied extensively. Roll et al., reported strong pre-treatment skin positivity with significant decline after VIT, although some patients maintained similar intradermal end-point concentrations, suggesting persistent underlying sensitivity (36). Pasaoglu et al., likewise observed decreased reactivity after VIT but noted continuing discordance between skin test results and clinical protection, confirming that declining cutaneous reactivity does not automatically equate to functional tolerance (37). Albanesi et al., in a 13-year real-life study, found parallel reductions in skin reactivity and serum IgE but showed that neither predicted protection reliably, reinforcing that skin tests are diagnostic rather than monitoring tools (38).

High-exposure populations add further complexity. Münstedt et al., observed that in beekeepers, live-sting provocation sometimes confirmed allergy when skin tests were negative, particularly in individuals with high natural tolerance or repeated low-dose exposure, indicating that real-life responses may not correspond with *in vivo* testing (5). Fehr et al., showed that the predictive value of skin tests varied with venom type and patient phenotype, and that negative tests did not exclude clinically important allergy, especially in patients tested long after the sting or with milder sensitization (17).

Age, timing, allergen concentration and methodology also shape interpretation. Carballada et al., demonstrated that older patients required lower intradermal concentrations to elicit reactions and that sensitivity fluctuated over time, limiting the prognostic utility of intradermal testing (39). In a pediatric cohort, Visitsunthorn et al., found lower sensitivity for prick than intradermal testing and showed that early negative tests often became positive after several weeks, confirming the importance of optimal timing (40).

Contemporary reviews emphasize standardized technique and combined interpretation. Jakob et al., stressed use of validated extract concentrations and integration of skin test results with serology, especially when testing soon after a sting to avoid false negatives (26). Real-world analyses echo this: Cichocka-Jarosz et al., noted that although skin tests have high diagnostic value when performed correctly, non-standardized concentrations or absent serologic evaluation reduce accuracy (41). Hollstein et al., showed that baseline skin test results did not predict relapse after VIT discontinuation and that clinical factors such as mast-cell disorders or severe initial reactions were more informative (42).

New allergen extracts have been introduced. Morales-Palacios et al., reported high diagnostic sensitivity and specificity for a novel venom extract but cautioned that interpretation still requires correlation with history, serology and exposure profile (43). Overall, skin prick and intradermal tests remain indispensable for diagnosing Hymenoptera venom allergy, yet their predictive and monitoring value is constrained by biological variability, extract composition, timing, patient phenotype and methodological factors. Clinical decisions about risk assessment and VIT continuation should not rely solely on these markers. Optimal application requires integration with clinical history, serologic testing and, when necessary, real-life sting outcomes.

### Total immunoglobulin E in Hymenoptera venom allergy: role and limitations

Total IgE has long been measured in Hymenoptera venom–allergic patients, but extensive evidence indicates minimal diagnostic or prognostic utility. Early attempts to use total IgE as a global indicator of allergic predisposition showed that it mainly reflects overall atopic load rather than venom-specific sensitization or sting-reaction risk. Large pediatric cohorts illustrate this: polysensitized children display higher total IgE and more eosinophilia than monosensitized peers, yet total IgE, eosinophils and age cannot distinguish these groups without specific IgE profiling (44). Thus, total IgE is heavily influenced by generalized atopy and unrelated exposures, limiting its relevance in venom allergy.

Hymenoptera-specific studies likewise show substantial overlap of total IgE values between patients with local and systemic reactions, with no consistent ability to predict severe sting reactions or systemic events during VIT. Longitudinal data indicate no correlation between baseline total IgE and VIT-related systemic reactions or clinical protection, and changes in total IgE during treatment do not reliably track the acquisition of tolerance (36). Hollstein et al., reported that patients with the most severe vespid sting anaphylaxis (Mueller grade IV) more often exhibited low total IgE than elevated levels, supporting the view that total IgE does not reflect reaction severity or phenotype (42). Overall, atopic status appears unrelated to anaphylaxis severity (45–47) except in specific high-exposure groups such as beekeepers (48). Although higher total IgE (e.g., >250 kU/L) is more often seen in patients with milder (grade I–II) reactions, this association is likely confounded by age and comorbidities (cardiovascular disease, raised baseline tryptase) that both lower IgE and increase risk of severe events (46, 49). Because total IgE tends to parallel venom-specific IgE, low total IgE can mask specific sensitization, so such patients warrant thorough clinical evaluation and, if indicated, repeat or alternative testing to avoid false-negative serology (47).

Total IgE may also be deceptively low in high-risk patients. Those with systemic mastocytosis or clonal mast-cell disorders frequently have very low circulating IgE despite clinically relevant venom allergy, likely because IgE is sequestered on expanded mast-cell surfaces rather than in serum. Low total IgE therefore cannot exclude sensitization or imply reduced risk (155). Conversely, markedly elevated total IgE often reflects polysensitization to aero- or food allergens instead of venom-specific immunity.

Multiple physiologic, hormonal and pharmacologic factors modify total IgE independently of venom allergy. In a patient undergoing *in-vitro* fertilisation, gonadotropin stimulation induced large fluctuations in venom-related IgE binding, while hCG caused a rapid decline despite no new stings or clinical change, showing that endocrine shifts can transiently alter IgE levels (50). Anti-IgE therapy such as omalizumab similarly raises measured total IgE several-fold via immune-complex formation, but these increases do not indicate heightened risk or better protection (51).

Collectively, these data show that total IgE should neither be used as a diagnostic test for venom allergy nor as a predictor of reaction severity, sting-challenge outcomes or VIT tolerance. It functions mainly as a nonspecific marker of atopic background and provides little venom-specific risk information. Interpretation of total IgE must always occur alongside clinical history and venom-specific diagnostics, with the understanding that neither low nor high values have direct implications for Hymenoptera venom allergy.

### Serologic detection of venom-specific IgE and component-resolved approaches

Specific IgE (sIgE) measurement is a key diagnostic tool in Hymenoptera venom allergy, complementing history and skin testing by documenting IgE-mediated sensitization to particular venoms or components. Early immunoenzymatic methods such as RAST and immunoblotting showed that venom-specific IgE responses vary widely among individuals and that qualitative allergen recognition patterns do not predict clinical severity. In 47 bee-sensitive subjects, immunoblotting identified IgE binding to multiple honeybee proteins, including phospholipase A_2_, hyaluronidase and acid phosphatase, yet no association with reaction severity emerged. Concordance with RAST was strongest in highly positive sera, whereas low-level RAST positivity often lacked visible bands, reflecting immunoblot's lower sensitivity and focus on linear epitopes compared with conformational epitopes captured by RAST (52). Case reports demonstrate how sIgE quantification clarifies unusual reactions: markedly elevated sIgE and IgG₄ in an Ectomomyrmex ant–allergic patient confirmed species-specific sensitization (53), and positive wasp venom sIgE supported an IgE-mediated mechanism in a patient with sting-induced cerebral infarction (156).

The natural history of venom-specific IgE has been studied in asymptomatic occupational cohorts. Among 72 Japanese pest-control operators, about one-third had positive sIgE, which generally declined over time and often became negative within several years, though persistence beyond 10 years occurred in a minority. Time since last sting and total IgE were the main determinants of sIgE kinetics, whereas age, number of stings and insect species had less impact. Once sIgE became negative, re-sensitization in the absence of new stings was rare, suggesting that serial sIgE monitoring reflects natural desensitization and helps interpret isolated positive results or VIT decisions (54). In highly exposed outdoor workers, sIgE positivity correlated with cumulative exposure rather than reaction severity, reiterating that sensitization alone does not equal clinical allergy and must be interpreted in epidemiologic context (7, 13).

Quantitative sIgE levels have limited ability to predict individual risk. Titers often remain elevated after severe reactions or during VIT despite clinical protection. In a near-fatal reaction during VIT, honeybee and Polistes sIgE remained high, illustrating that positive sIgE does not preclude tolerance (31). Carballada et al., observed significant declines in sIgE during VIT, sometimes to undetectable levels, yet patients with persistent positivity frequently remained protected, whereas mild systemic reactions could still occur in those with low sIgE. They concluded that sIgE changes document immunologic modulation but are not sufficient for assessing protection (39). Similar patterns were seen in large Vespula VIT cohorts, where sIgE decreased during and after therapy, but complete negativization was rare and did not clearly align with long-term sting tolerance (38, 55).

In selected scenarios, sIgE levels and component-specific patterns support risk stratification. In bee-sting anaphylaxis, elevated sIgE to Api m 4 identified a phenotype with more severe index reactions, broader sensitization (including Api m 1), more systemic reactions during VIT and slightly reduced sting-challenge protection. During VIT, Api m 1 and Api m 4 sIgE declined markedly, but intradermal sensitivity changed little, underscoring the complex relationship between serologic markers and clinical reactivity (56). In a large pediatric ultrarush VIT protocol, very high baseline bee-venom sIgE predicted systemic reactions during build-up with useful sensitivity and specificity, whereas no equivalent cut-off was found in adults, suggesting age-dependent differences in prognostic value (57). Baseline sIgE generally does not predict long-term relapse risk after VIT discontinuation (38).

Comparative analyses of sIgE platforms highlight technical considerations. Pfender et al., showed that a rapid lateral-flow assay (ALFA) had high sensitivity and specificity for bee and wasp venom with good agreement with ImmunoCAP and ALLERG-O-LIQ, but fewer false positives in highly atopic controls, suggesting nonspecific binding on some solid-phase platforms (58). Watanabe et al., found that IMMULITE 3gAllergy had higher sensitivity and slightly better specificity than ImmunoCAP in a Japanese cohort, though both systems produced positive results in sting-naïve subjects, likely owing to CCDs or other cross-reactive epitopes (59). Marquès et al., reported only moderate concordance between ImmunoCAP and Euroline for molecular diagnostics; ImmunoCAP provided quantitative monitoring, whereas Euroline offered broader semi-quantitative profiles but tended to suggest more dual-VIT indications, illustrating how assay choice influences clinical conclusions (60).

Component-resolved diagnostics (CRD) have substantially improved sIgE interpretation. Recombinant allergens for Vespula (Ves v 1, Ves v 5), Polistes (Pol d 1, Pol d 5) and honeybee (Api m 1, 2, 3, 4, 5, 10 and others) increase sensitivity and enhance species discrimination (Table 1). Korošec et al., showed that combined rVes v 5 and rVes v 1 identified Vespula allergy with about 92% sensitivity (61). Köhler et al., found that adding recombinant Api m 2, 3, 4, 5 and 10 to Api m 1 increased honeybee diagnostic sensitivity to 94% and revealed 39 distinct sensitization profiles, reducing false double-positivity to wasp venom (62). Further studies confirm that use of multiple components, particularly Api m 1 and Api m 10, significantly improves diagnostic yield, especially in highly exposed individuals (63, 64).

**Table 1:** Component-resolved diagnostics (CRD) in Hymenoptera venom allergy.

Species	Allergen	Type	Diagnostic role	Cross-reactivity/limitations
Apis mellifera	Api m 1 (PLA2)	Species-specific marker	Primary marker of genuine bee sensitization	Low cross-reactivity
	Api m 3	Supportive marker	Associated with broader sensitization	Limited cross-reactivity
	Api m 4	Supportive marker	Linked to more severe reactions	Limited cross-reactivity
	Api m 10 (icarapin)	Key marker allergen	Improves sensitivity, especially when Api m 1 negative	Labile; may be underdetected
	Api m 2, Api m 5	Cross-reactive allergens	Increase diagnostic sensitivity	Cross-react with vespid homologues (e.g., Ves v 3, Pol d 3)
Vespula spp.	Ves v 5 (antigen 5)	Species-specific marker	Highly sensitive and specific for Vespula allergy	Limited cross-reactivity with Apis
	Ves v 1 (PLA1)	Species-specific marker	Complements Ves v 5; improves diagnostic sensitivity	Low cross-reactivity
	Ves v 3 (DPP IV)	Cross-reactive allergen	Contributes to sensitization profile	Cross-reacts with Api m 5, Pol d 3
Polistes dominula	Pol d 5 (antigen 5)	Species-related marker	Useful marker within vespids	Strong cross-reactivity with Ves v 5
	Pol d 1 (PLA1)	Species-related marker	Supports diagnosis	Cross-reactivity with other vespids
	Pol d 3 (DPP IV)	Cross-reactive allergen	May assist BAT interpretation	Cross-reacts with Api m 5, Ves v 3
Vespa velutina	Ves v 5 (antigen 5 homolog)	Putative marker	Potential marker for species identification	Strong cross-reactivity with Ves v 5 and Pol d 5
	Ves v 1 (PLA1 homolog)	Putative marker	Possible diagnostic relevance	Limited specificity vs. other vespids
	Other homologous proteins	Cross-reactive allergens	Limited current CRD availability	Significant overlap with Vespula and Polistes

New allergens and proteomic data continue to expand the molecular toolkit. Icarapin (Api m 10) was identified as a major bee-venom allergen recognized by most allergic patients, while recombinant studies of Polistes and Vespula components, including DPP IV proteins such as Pol d 3, showed extensive cross-reactivity with homologous Api m 5 and Ves v 3. BAT using these recombinant DPP IV allergens, mirrors sIgE and skin-test patterns and may help resolve primary sensitization in double-positive patients (65–67). Reviews summarize that marker allergens (Api m 1, 3, 4, 10; Ves v 1, Ves v 5; Pol d 1, Pol d 5) permit discrimination between primary bee and vespid allergy, whereas cross-reactive glycoproteins and pan-allergens frequently underlie polysensitization and may require CAP-inhibition or BAT for clarification (26, 68, 69).

Distinguishing true double sensitization from cross-reactivity remains difficult. Savi et al., showed that component testing alone would have suggested dual VIT in many double-positive patients, whereas CAP-inhibition substantially reduced the proportion classified as truly double-sensitized (70). Multiple studies confirm its superiority over singleplex tests and molecular diagnostics in differentiating Vespula vs. Polistes sensitization, reporting the lowest frequency of true double sensitizations (≈25%) among tested methods (71, 72). CAP-inhibition has proven clinically useful: in one series it allowed 31 of 45 double-vaccinated patients to receive only a single VIT, and case reports show it can resolve ambiguous results when molecular diagnostics fail (71, 73). However, CAP-inhibition is limited to specialized centers, is costly, and can yield results that are sometimes difficult to interpret (27). Technical complexity and expense therefore restrict routine use despite potential downstream savings from avoiding unnecessary double-VIT (72).

Jovanovic et al., demonstrated that CCD-free markers such as Api m 1 and Ves v 5 have high specificity and acceptable sensitivity, limiting overdiagnosis associated with whole-venom extracts (157). Tischler et al., proposed a ≥5:1 dominance ratio of sIgE to one venom or its marker allergen to designate the likely culprit and support single-venom VIT, reserving CRD and BAT for cases with balanced sensitization (74). Large cohorts confirm that asymptomatic sensitization to both extracts and marker allergens is common and that double positivity is not synonymous with true double allergy (13, 18, 75).

Guidelines integrate these insights. The German S2k guideline recommends venom-specific sIgE measurement at first presentation, repeating tests after several weeks if initially negative, and discourages indiscriminate screening because 23%–40% of adults and up to half of children have venom IgE without systemic reactions. For double-positive or implausible constellations, CCD screening and component testing with Api m 1, 2, 3, 4, 10 and Ves v 1, Ves v 5 are advised (29). Expert systems such as Allergenius® show how structured interpretation of microarray-based profiles, including CCD markers, can distinguish true venom allergy from cross-reactivity and align molecular findings with clinical history and skin tests (76). Recent reviews reiterate that sIgE is essential to confirm IgE-mediated venom allergy but that values and patterns must always be interpreted in clinical context, with awareness of asymptomatic sensitization, CCD effects, cross-reactivity and platform-specific artefacts (75, 77).

Overall, immunoenzymatic testing of venom-specific IgE, particularly when extended to molecular components and interpreted alongside history and skin tests, is indispensable for diagnosing Hymenoptera venom allergy and selecting appropriate venom for immunotherapy. However, neither sIgE presence nor magnitude reliably predicts reaction severity, long-term protection or relapse, and positive results are frequent in asymptomatic or polysensitized individuals. Current evidence supports using sIgE primarily as a sensitive indicator of sensitization, with CRD, CAP-inhibition, CCD assessment and, when needed, BAT to refine diagnosis and guide personalized management.

### Cross-reactive carbohydrate determinants: impact on venom allergy diagnostics

Cross-reactive carbohydrate determinants (CCDs) are glycan epitopes on many plant and invertebrate glycoproteins, including Hymenoptera venom allergens, and can induce IgE that binds broadly across unrelated proteins. Rendić et al., characterized immunogenic CCD structures, particularly N-glycans with core α1,3-fucosylation, β1,2-xylosylation and Lewis-type antennal motifs, and showed that the corresponding glycosyltransferases are highly expressed in venom glands. This explains why many venom enzymes, such as hyaluronidases and phospholipases, carry CCDs and why sera from venom-allergic patients frequently recognize CCD-bearing glycoproteins from both venoms and plants. Because CCD-IgE targets carbohydrate rather than protein epitopes, it produces extensive *in vitro* cross-reactivity without matching clinical relevance, supporting use of recombinant nonglycosylated allergens or CCD-blocking approaches to define true sensitization (78).

Clinical studies quantify the diagnostic impact of CCD-IgE. Carballada et al., found that double positivity to honeybee and wasp extracts was common and strongly associated with elevated total IgE, false-positive multiallergen screening and high alcohol intake. Many double-positive patients had detectable CCD-IgE, and CCD inhibition frequently revealed mono-sensitization consistent with history, indicating that apparent bee- and wasp-double allergy is often CCD-driven (39). In a hospital cohort of heavy drinkers, Alvela-Suarez et al., similarly observed frequent CCD-IgE with broad, clinically irrelevant venom positivity in individuals without sting anaphylaxis, inflating apparent multi-venom sensitization (18).

The functional relevance of CCD-IgE has been evaluated with BAT. Kaulfürst-Soboll et al., showed that CCD-IgE can activate basophils *in vitro* when stimulated with CCD-rich glycoproteins, but these responses did not correlate well with clinical symptoms, suggesting limited clinical impact and underscoring the value of CCD-free or recombinant allergens for diagnostic specificity (79). Sturm et al., combined BAT, Western blot inhibition and CRD (recombinant Api m 1, Api m 2, Ves v 1, Ves v 2, Ves v 5) in patients double-positive to bee and vespid venom. They found that CCD-mediated binding accounted for much of the multiple serologic positivity, whereas true double sensitization was confirmed in only a minority; BAT and CRD most closely matched sting histories (80).

Reviews and mechanistic papers show that CCD responses are frequent and broadly distributed. Treudler estimated that about one-fifth of atopic individuals have CCD-IgE, which can cause false-positive *in vitro* tests to numerous plant and insect allergens, including venoms. Although some carbohydrate epitopes such as α-Gal are clinically relevant, classical CCDs generally represent subclinical sensitization and require CRD for proper interpretation (69). Gattinger et al., engineered recombinant CCD neo-epitopes as standardized tools for quantifying CCD-IgE and selectively blocking carbohydrate-driven signals, thereby clarifying underlying protein-specific sensitization profiles (81).

Proteomic and venom-focused studies further illustrate CCD impact. Popescu et al., noted that many native venom enzymes are heavily glycosylated and responsible for the broad IgE reactivity seen in extract-based tests. They advocate systematic CCD screening and routine use of CCD-free recombinant allergens to distinguish CCD-mediated cross-reactivity from clinically meaningful sensitization, while acknowledging that protein homology may also drive cross-reactivity (68). Abram et al., detected CCD-IgE in nearly half of 51 venom-allergic patients; CCD inhibition selectively removed multiple glycoprotein bands on immunoblot but spared binding to nonglycosylated recombinant phospholipase and antigen 5, resolving pseudo-multi-venom sensitization and revealing the true culprit insect (82).

Large observational cohorts document population-level effects. Bergmann-Hug et al., found that venom sIgE and CCD-marker positivity were frequent in both venom-allergic patients and controls, especially pollen-allergic individuals with high total IgE, but sIgE levels were generally low, with sIgE/total IgE ratios <0.1, suggesting limited clinical relevance. CCD positivity inflated apparent multi-venom sensitization in people without systemic sting reactions, emphasizing the risk of over-interpretation (83). In venom-allergic patients undergoing VIT, Jovanovic et al., showed that CCD-IgE and multiple extract positivity were common, but CCD inhibition and component testing with rApi m 1, rApi m 10, rVes v 5 and others substantially reduced the number classified as truly multi-sensitized. True multiple sensitizations, defined by IgE to at least one marker component of each species, was much less frequent; combining CCD testing, recombinant components and inhibition assays was critical for selecting the appropriate venom(s) for VIT (84).

Overall, CCD-specific IgE is common in venom-allergic and atopic populations and can activate basophils *in vitro* but is usually of low clinical relevance. Its main effect is diagnostic: it inflates apparent venom sensitization and double positivity, complicating distinction between mono- and double sensitization when only extract-based assays are used. Modern diagnostic strategies therefore integrate CCD markers, CCD-blocking reagents, CCD-free recombinant allergens, CRD and, when indicated, BAT to distinguish clinically meaningful sensitization from carbohydrate-driven cross-reactivity and to guide accurate VIT selection.

### Venom-specific IgG4 and IgE-blocking activity: markers of immune modulation

IgG4 antibodies induced during allergen exposure and immunotherapy are often viewed as “blocking antibodies” that can compete with IgE for allergen binding and thereby limit effector-cell activation. However, Hymenoptera venom studies show that although venom-specific IgG4 is robustly induced by VIT and repeated natural stings, absolute IgG4 titers alone do not reliably predict clinical protection. Early work by Wolf showed that higher venom-specific IgG (>3.5 μg/mL) correlated with fewer systemic reactions after re-stings, yet systemic events still occurred in some patients, indicating that IgG4 levels alone are insufficient for risk estimation (31).

Multiple VIT cohorts have examined IgE and IgG4 kinetics. A prospective study demonstrated a significant early fall in venom-specific IgE and a sustained rise in IgG4 over 24 months, confirming strong IgG4 induction but showing that serologic changes could not, by themselves, identify protected patients (85). Pasaoglu et al., similarly observed increasing IgG4 and decreasing IgE during VIT, yet systemic reactions still occurred, indicating that IgG4 reflects immunologic modulation rather than guaranteed tolerance (37). In a retrospective cohort of 56 bee- or wasp-allergic patients, Spoerl et al., reported marked IgG4 increases, more pronounced in wasp VIT, and IgE reductions, but no IgG4 threshold predicted sting-challenge outcomes, reinforcing IgG4's role as a mechanistic rather than prognostic marker (86).

Long-term data support these findings. In a 13-year Vespula VIT study, Albanesi et al., documented substantial IgG4 rises during the 5-year treatment and gradual declines afterward, paralleling reductions in circulating and skin-bound IgE. However, IgG4 trajectories and IgG4/IgE ratios did not consistently match sting outcomes; many patients remained protected despite falling IgG4, and some showed discordant immunologic and clinical profiles. The authors proposed that IgG4/IgE ratios may be more informative than either alone but remain insufficient to define long-term protection or to guide VIT discontinuation (38). In another series of 88 adults, higher venom-specific IgG4 correlated with lower skin test reactivity, suggesting functional blocking at the mast-cell level, but no validated protective threshold could be defined (87).

Population studies and tolerance models clarify IgG4's broader role. In 274 Japanese subjects with diverse sting histories, Hayashi et al., found sIgG4 positivity common, with patterns such as sIgG4+/sIgE− and sIgG4−/sIgE+, indicating partially independent pathways. No clear relationship emerged between sIgG4 levels and systemic sting reactions, suggesting that IgG4 induction alone does not ensure suppression of IgE-mediated symptoms and that clinical history remains more informative (7). In a Spanish beekeeper survey, venom-specific IgG4 was much higher in frequently stung, tolerant individuals than in controls, supporting an association with tolerance, yet no strict IgG4 cut-off separated tolerant from reactive beekeepers (158). Ruiz-León et al., similarly found that tolerant beekeepers had elevated IgG4 and distinctive regulatory T-cell signatures compared with allergic patients, but concluded that IgG4 is part of a tolerance-associated profile rather than a stand-alone biomarker (75).

Component-resolved analyses provide further nuance. Blank et al., measured IgE and IgG4 against whole venoms and major components (Api m 1, Api m 10, Ves v 1, Ves v 5) in VIT patients and non-allergic beekeepers, documenting strong allergen-specific IgG4 induction during VIT and robust IgG4 responses in naturally tolerant beekeepers with high sting frequencies. IgG4/IgE ratios shifted in favour of IgG4 for particular components, yet tolerance occurred across heterogeneous IgG4 profiles, indicating that IgG4 reflects but does not solely drive immune tolerance (77).

Functional assays probing IgG4-mediated IgE blocking add mechanistic support. A long-term single-centre study found that most patients after ≥5 years of VIT exhibited measurable IgE-blocking activity, which correlated with venom-specific IgG4; low or absent blocking associated with less favourable immunologic profiles and possible relapse risk. The authors suggested that combined measurement of IgG4 and blocking activity may be more informative than IgG4 alone, but current data do not justify using these parameters to decide on VIT discontinuation or to predict long-term outcomes with high certainty (88).

In summary, venom-specific IgG4 and related blocking activity provide valuable insight into immune deviation and tolerance induction. IgG4 rises during VIT and in frequently stung, tolerant individuals, usually accompanies reductions in IgE and skin reactivity and forms part of a tolerance-associated immune signature. However, absolute IgG4 concentrations, even when high, do not reliably predict protection, relapse risk or suitability for stopping VIT. IgG4 and IgG4/IgE ratios, especially when combined with functional IgE-blocking assays, appear promising adjunctive markers but must be interpreted together with clinical history, sting-challenge outcomes, skin tests and other parameters, rather than used as isolated predictors.

### Serum tryptase as a biomarker of mast-cell burden and anaphylaxis risk

Tryptase, a mast-cell–derived serine protease, is a central biochemical marker of acute anaphylaxis and mast-cell burden in Hymenoptera venom allergy. An early clinical example was a near-fatal anaphylaxis during maintenance VIT, where serum tryptase measured 2 h after the event reached 37.5 µg/mL (normal: 5.6–13.5 µg/mL), confirming massive mast-cell degranulation and distinguishing the reaction from vasovagal or toxic events; acute-phase tryptase has since become standard in evaluating suspected anaphylaxis (31).

Basal serum tryptase (BST) has been extensively investigated as a predictor of sting-reaction severity and a marker of occult mast-cell disease. In 109 venom-allergic patients with systemic sting reactions, Kucharewicz et al., found elevated BST (>11.4 µg/L) in 11% and rising median values across Mueller grades, with significantly higher levels in grade IV reactions. BST correlated with age and was higher in wasp- than bee-allergic patients, leading the authors to propose BST as a quantitative risk marker even at modest elevation (89). Potier et al., in 140 patients with Hymenoptera- or Diptera-induced anaphylaxis, reported that elevated BST (≥13.5 ng/mL) associated with more severe reactions and a phenotype dominated by flushing with few urticarial lesions; about one-third had unrecognized mastocytosis. They recommended BST assessment in severe sting reactions, especially where flushing predominates and urticaria is sparse (90).

However, normal BST does not exclude severe reactions. Gomis described a patient with recurrent systemic anaphylaxis during bee VIT and normal BST (2.2 µg/L), illustrating that neither sIgE nor tryptase reliably predicts VIT safety (51). Galera et al., similarly reported repeated cardiovascular reactions during rush VIT in a patient with normal BST (2.4 µg/L); tryptase aided in ruling out systemic mastocytosis but did not prevent severe reactions, showing that normal BST does not guarantee uneventful therapy (91).

Pediatric data show that even relatively low tryptase values can indicate increased mast-cell activity. In 19 children undergoing rush VIT, Cichocka-Jarosz et al., found that higher baseline tryptase predicted systemic reactions and correlated with severity, while PGD_2_ metabolites had limited predictive value (92). A subsequent case-control study showed significantly higher BST in venom-allergic than healthy children, with an AUC of 0.70 at a 2.57 ng/mL cut-off, suggesting that even modest elevations may reflect increased mast-cell burden in pediatric systemic sting reactions (3).

Adult cohorts highlight BST's dual role as risk marker and disease indicator. In 489 adults (venom-allergic patients, beekeepers and controls), Carballada et al., found slightly higher BST in both allergic patients and beekeepers compared with controls, independent of age and atopy, though only a minority exceeded the upper normal limit; BST did not correlate with severity of previous systemic reactions (93). Elevated BST should nonetheless prompt evaluation for clonal mast-cell disease, as illustrated by Gülen et al., who reported three wasp-allergic adults with BST 12–23 µg/L, all undergoing bone marrow biopsy for suspected mastocytosis (94). Pastorello et al., described two older men with severe sting anaphylaxis and very high tryptase (52.8 and 153 ng/mL) ultimately attributed to abdominal aortic aneurysms; tryptase normalized after surgical repair, demonstrating that markedly elevated BST may reflect non-allergic conditions involving mast-cell accumulation (95).

Prospective VIT cohorts refine the prognostic value of BST. Vega-Castro et al., measured baseline tryptase in 150 patients starting VIT and identified clonal mast-cell disease in a small subset after bone marrow biopsy, yet BST did not differ significantly between patients with and without systemic reactions during VIT; standard cut-offs (5 or 11.4 µg/L) lacked predictive power (96). Farioli et al., in 365 venom-allergic patients, found that mean BST was significantly higher in those with severe (grade III–IV) reactions than in those with milder reactions; the proportion of severe reactions increased from about one-third in patients with BST <4 ng/mL to almost 90% in those with BST >7.5 ng/mL. Each 1 ng/mL increase in BST raised the odds of a severe reaction by about 46%, supporting BST as a strong severity risk marker and suggesting clinically relevant intermediate thresholds around 7 ng/mL (97).

Population-based data reveal limitations of tryptase as a screening test. In 257 unreferred hunters and fishers, Zink et al., observed a low mean BST (3.74 ± 3.48 µg/L), only 3.5% with elevated values above 11.4 µg/L, and minimal overlap between high BST and systemic sting reactions; only one of nine high-tryptase subjects reported anaphylaxis. In this high-exposure but largely asymptomatic cohort, BST was insensitive for identifying those at risk, and detailed sting history plus targeted IgE testing remained key tools (13).

In acute anaphylaxis, tryptase has been compared with other biomarkers. Korošec et al., reported that circulating basophil counts, FCER1A/CPA3/HDC expression and serum CCL2 discriminated anaphylaxis from controls better than tryptase or basophil activation, although acute tryptase >11.4 µg/L still occurred in ∼71% of cases, supporting its ongoing diagnostic role while suggesting added value of cellular and chemokine markers (98).

Reviews and consensus documents summarize evolving concepts. Platzgummer et al., describe tryptase as a stable marker with an upper 95th percentile around 11.4 µg/L, measured by ImmunoCAP, and recommend sampling 30 min to 3 h after symptom onset and again after ≥24 h, applying the “20% + 2” rule (acute ≥ baseline × 1.2 + 2 µg/L) to confirm mast-cell activation. They advise baseline tryptase measurement in all patients with systemic sting reactions and further work-up for clonal mast-cell disorders when elevation persists (99). O'Connell similarly presents BST as a marker of mast-cell burden and risk, stressing that hereditary α-tryptasemia (HαT), present in 3%–5% of Western populations, is a common cause of elevated BST independent of mastocytosis. Acute/baseline comparison using the 20% + 2 rule is highlighted as central to diagnosing anaphylaxis. A recent 2024 review by Oštrić Pavlović et al., updates the upper reference limit for BST to 8.4 µg/L and confirms that the 20% + 2 rule or acute-to-baseline ratio cut-offs offer high sensitivity and specificity for anaphylaxis (100).

Guidelines incorporate these insights. The German S2k guideline on honeybee and vespid venom allergy identifies elevated BST and systemic mastocytosis as the strongest predictors of severe sting anaphylaxis, noting that risk rises even at values below 11.4 µg/L. It recommends BST measurement in all patients with systemic reactions beyond the skin, repeat sampling when acute levels have been measured and referral for mastocytosis work-up when BST persistently exceeds 20 µg/L (29).

Real-world VIT cohorts illustrate how tryptase interacts with other risk markers. In 480 venom-allergic patients, Fehr et al., found no association between whole venom or component-specific IgE and reaction severity, whereas BST, particularly in wasp-allergic patients, correlated with severity and was more frequently elevated. They concluded that elevated BST, especially in older patients with short latency to symptoms and absent skin signs, is a more informative marker than IgE for identifying those at high risk and should be integrated into multifactorial risk assessment (17).

Overall, across pediatric and adult cohorts, case reports, population studies, reviews and guidelines, tryptase emerges as a crucial biomarker in Hymenoptera venom allergy. Acute tryptase supports the diagnosis of sting-induced anaphylaxis and helps distinguish true mast-cell activation from mimicking conditions, while BST identifies patients with increased mast-cell burden who are at higher risk of severe reactions and may harbour clonal mast-cell disorders or hereditary α-tryptasemia. Nevertheless, normal BST does not guarantee mild reactions or safe VIT, and elevated levels must be interpreted within the broader clinical and diagnostic context.

### Basophil biology and basophil activation testing in venom allergy

Basophils, although the least abundant myeloid leukocytes, play a major role in IgE-mediated hypersensitivity. They rapidly migrate into inflamed skin and mucosa, release histamine and other mediators and secrete IL-4 and IL-13, acting both as effector cells and immune modulators. Their ready accessibility in peripheral blood makes them attractive surrogate cells for studying IgE-dependent activation and for functional diagnostics in Hymenoptera venom allergy (101). Experimental models of cutaneous basophil hypersensitivity show that IgE-sensitized basophils home to allergen-exposed skin and participate in delayed-type inflammation, underscoring their *in vivo* relevance (102).

Functional testing exploits basophils' capacity to respond to IgE-dependent and IgE-independent stimuli. Studies in which basophils from allergic and non-allergic donors are exposed to whole venoms, purified allergens, anti-IgE or T-cell–dependent stimuli show robust, dose-dependent activation in sensitized individuals but minimal responses in non-sensitized donors. Basophils can also respond to T-cell–only antigens via indirect mechanisms, highlighting their tight linkage with adaptive immunity and providing a rationale for basophil-based assays in venom allergy (35).

Basophil activation testing (BAT) quantifies allergen-induced activation in whole blood, typically using flow-cytometric markers such as CD63 and CD203c. CD63 reflects degranulation through granule–membrane fusion, while CD203c is a surface molecule rapidly upregulated upon IgE-dependent stimulation. Methodological studies have optimized stimulation conditions (including IL-3 priming), marker combinations (CD63, CD203c, CD123, HLA-DR, CCR3) and gating strategies to distinguish basophils from eosinophils and other leukocytes. Pre-analytical variables, including anticoagulant type, time to processing and storage conditions, markedly influence basophil responsiveness, reinforcing the need for standardization (103, 104).

Clinically, BAT is regarded as an adjunctive tool, especially when conventional tests are inconclusive. Reviews describe BAT as a second-line method that can improve specificity, reduce the need for sting provocation and support diagnosis when skin tests or serum IgE are negative or equivocal despite a strong history. A positive BAT indicates functional sensitization; a negative BAT in sensitized individuals may suggest low effector-cell responsiveness or waning clinical relevance. BAT shows high negative predictive value in borderline situations and is particularly useful in complex cases such as double sensitization or when skin testing is contraindicated (105, 106).

Several clinical studies confirm BAT's diagnostic utility. In adults with systemic wasp allergy and controls, Nullens et al., showed that >15% CD63-positive basophils clearly discriminated allergic from non-allergic subjects. Basophil responsiveness decreased during VIT but rarely normalized fully and did not consistently predict protection, indicating that BAT is more useful diagnostically than prognostically (107). Ebo et al., demonstrated that BAT aided in identifying the culprit venom in double-sensitized patients, refining VIT selection when skin tests and sIgE were insufficiently discriminatory (108).

BAT has also been evaluated in systemic mastocytosis and other special populations. In clonal mast-cell disorders, basophils often show strong venom-induced activation despite altered mast-cell biology, helping confirm clinically relevant allergy, although negative BAT does not exclude severe reactions (109, 107). In naturally exposed Japanese beekeepers, tolerant individuals frequently displayed lower basophil reactivity, but overlap with allergic individuals limited predictive accuracy for systemic reactions (110).

Methodological work has identified key limitations. Depince-Berger et al., optimized an indirect BAT using healthy donor basophils incubated with patient sera and showed that serum factors (including IgE and other immunoglobulins) and donor variability strongly influence responses, necessitating controlled conditions and precise gating that distinguishes basophils from B cells and other populations (159). Kim et al., reported that IL-3 priming increases sensitivity but also nonspecific activation, and that different basophil-gating strategies (CCR3-based vs. CD123/HLA-DR–based) capture distinct basophil pools, affecting comparability across laboratories (103). Studies in chronic spontaneous urticaria and food allergy similarly show disease-specific activation patterns, indicating that BAT cut-offs cannot be extrapolated between conditions (101).

Guidelines now define BAT's role. The German S2k guideline on honeybee and vespid venom allergy states that BAT using CD63/CD203c is useful when clinical suspicion is strong but both skin testing and sIgE are negative or ambiguous. It recommends performing BAT only in experienced laboratories with validated protocols and interpreting results alongside clinical history and other tests; BAT is not recommended as a screening tool in asymptomatic sensitization, and sting provocation remains the gold standard for assessing protection (29). Recent reviews highlight BAT's usefulness in double positivity, mastocytosis, hereditary α-tryptasemia and situations where skin testing is contraindicated, while noting limited availability, costs and lack of universal standardization as barriers to wider use (105).

In summary, basophils play a key role in the effector phase of Hymenoptera venom allergy and offer a unique insight into IgE-mediated activation. BAT, using markers such as CD63 and CD203c, can identify allergen-specific reactivity, help clarify diagnosis when conventional tests are inconclusive, and guide venom choice in complex sensitization cases. However, BAT performance is highly dependent on technical factors, and the absolute percentages of activated basophils or marker levels are not validated indicators of sting-reaction risk or venom immunotherapy (VIT) success. Additionally, although BAT can show changes in basophil responsiveness during venom immunotherapy, it has not been validated as a reliable biomarker of long-term protection or sustained tolerance after stopping VIT. Current evidence suggests that BAT results do not reliably predict relapse risk or clinical outcomes post-therapy and should not be used for prognostic decisions. Nonetheless, BAT is a valuable adjunctive test, best applied in specific clinical situations and interpreted alongside detailed patient history, skin tests, serum IgE, and mast-cell–related markers like basal tryptase. Because BAT remains a highly specialized technique primarily available at expert centers with advanced flow cytometry capabilities and validated protocols, its routine use is limited. Patients with complex diagnostic profiles may need referral to specialized allergy centers for BAT assessment. In most clinical settings, diagnosis continues to depend on clinical history, skin testing, serum-specific IgE, and, when available, component-resolved diagnostics.

### Histamine release and mast-cell–based functional assays

Histamine release from mast cells and basophils is a key early event in IgE-mediated Hymenoptera venom allergy. Early functional assays based on histamine liberation were among the first *in vitro* tests to demonstrate allergen-specific effector-cell activation. Sobotka et al., showed that purified venoms induced highly sensitive and specific histamine release: 13/16 venom-allergic patients displayed >50% release at 0.001–1.0 µg/mL, whereas none of 12 non-allergic controls responded even at 10 µg/mL, and reactions were largely species-specific. These findings established venom-induced basophil histamine release as a sensitive and specific indicator of insect-sting allergy and underlined the diagnostic advantage of using natural venoms (111).

Insights into histamine dynamics during allergic inflammation also come from cutaneous late-phase reaction models. Charlesworth et al., studied ragweed-allergic adults and observed substantial basophil (∼14-fold) and eosinophil (∼6-fold) influx into allergen-challenged skin, with even greater enrichment in blister fluid. Histamine showed a biphasic curve: an early peak around 13 ng/mL at 1 h, a decline to ∼2 ng/mL at 5–7 h and a second peak (∼9.8 ng/mL) at 12 h that correlated strongly with basophil accumulation (*r* = 0.81). PGD_2_ peaked early and then declined without a late increase. These data support a model in which mast cells dominate immediate-phase responses, while infiltrating basophils substantially contribute to late-phase histamine production and inflammation (112).

Recently, functional mast-cell–based diagnostics have been developed, analogous to BAT. Bahri et al., established a mast cell activation test (MAT) using human mast cells differentiated from CD117+/CD34+ progenitors, passively sensitized with patient sera and stimulated with allergen. Mast-cell degranulation was measured by CD63/CD107a upregulation and β-hexosaminidase and PGD_2_ release. In the Hymenoptera venom arm, mast cells sensitized with sera from wasp-allergic patients showed dose-dependent CD63 upregulation after wasp venom, whereas honeybee-only sera did not, confirming specificity. Although the principal clinical validation was in peanut allergy, where MAT outperformed BAT, sIgE, CRD and SPT (AUC 0.99 vs. 0.94–0.70), these data suggest MAT can detect clinically relevant venom-induced mast-cell activation (113).

Animal models provide direct mechanistic evidence that mast cells are indispensable for IgE-mediated anaphylaxis and histamine-dependent tissue responses. In mast-cell-deficient Cpa3-Cre; Mcl-1^fl/fl^ mice, mast cells were reduced by 92%–100% and basophils by 58%–78%. These animals showed almost no swelling or leukocyte influx during IgE-dependent passive cutaneous anaphylaxis, virtually absent chronic allergic skin inflammation and complete resistance to IgE-dependent passive systemic anaphylaxis. Local engraftment of *in vitro*–derived mast cells restored cutaneous responses, confirming mast cells as essential effector cells for both cutaneous and systemic anaphylactic reactions, with basophils providing only partial contributions (114).

Clinical translation has progressed further with LAD2-cell MAT. Koren et al., optimized LAD2 mast cells for IgE-mediated degranulation in venom allergy using IL-33, IL-6 and stem-cell factor pre-stimulation, significantly enhancing CD63 upregulation. In 39 venom-allergic patients and 22 controls, LAD2 MAT achieved an AUC of 0.67 with an optimal cut-off of 5.5% CD63+ mast cells; all controls were negative. LAD2 responses correlated with BAT (Rs = 0.48) and venom-specific IgE (Rs = 0.33), and patients with severe sting reactions (Mueller III–IV) showed significantly higher activation than those with mild/moderate reactions. Notably, in BAT non-responders, LAD2 MAT detected venom-specific reactivity in 54%, often identifying a single culprit venom in cases where other tests indicated double sensitization. The authors proposed LAD2 MAT as a complementary diagnostic tool, particularly valuable in BAT non-responders and potentially useful for severity assessment and VIT selection (115).

Mast-cell–related pathology intersects with clinical management of venom allergy in patients with clonal mast-cell disorders. Jarkvist et al., reported that Vespula-allergic adults with systemic mastocytosis or monoclonal mast-cell activation syndrome typically had elevated baseline tryptase (median: 18 ng/mL) and might show weak or atypical sensitization despite life-threatening anaphylaxis. Accurate diagnosis of clonal disease therefore requires integrated evaluation, including skin tests, venom-specific and component-specific IgE and bone-marrow examination, since mast-cell burden and reactivity strongly modify histamine-mediated risk and shape long-term VIT strategies (116).

In summary, histamine-release assays, cutaneous provocation models, mast-cell activation tests and mast-cell-deficient animal models collectively establish mast cells as central drivers of immediate and late-phase responses in Hymenoptera venom allergy. Early work (Sobotka) demonstrated allergen-specific histamine release; human provocation studies clarified phase-specific contributions of mast cells and basophils; modern MAT platforms detect clinically relevant mast-cell activation; and genetic ablation models confirm mast cells' indispensability in anaphylaxis.

### Controlled sting challenge with live insects: indications and risk–benefit profile

Provocation with a live insect or controlled sting challenge approximates real-life Hymenoptera exposure more closely than any other test and is widely regarded as the reference method for confirming clinical reactivity or protection, but it is also the highest-risk diagnostic tool. In one early prospective series of 55 patients with prior systemic reactions, in-hospital sting challenges reproduced systemic symptoms in a subset and allowed monitoring of cardiovascular parameters and endothelial activation markers such as von Willebrand factor, which rose markedly during reactions. These data showed that live-sting provocation can objectively document clinical reactivity but must be restricted to settings with full resuscitation capacity because of clear anaphylaxis risk (117).

Sting challenges are particularly important for assessing VIT effectiveness. In 74 patients who had completed standardized VIT, Golden et al., performed deliberate sting challenges to verify protection. Most were protected, but some still developed systemic reactions; these patients tended to have higher venom-specific IgE and greater post-challenge rises in serologic and clinical markers. The study demonstrated that tolerance to field or controlled stings cannot be inferred from serology alone and that sting challenge remains the only direct way to verify protection in selected patients (118). Similar results were obtained by Koschel et al., who performed controlled stings in 100 patients on maintenance VIT and found that a minority remained clinically reactive despite adequate dosing, emphasizing that VIT is highly, but not universally, protective and that provocation can reveal residual risk (119).

Case reports highlight the interpretive value of natural stings as “unplanned” provocation tests. Zidarn described a 66-year-old man who developed systemic anaphylaxis to a bee sting several years after completing VIT; repeated field stings appeared to act as immunologic “boosters,” maintaining high IgE and restoring clinical reactivity despite earlier desensitization, demonstrating that tolerance may wane over time (120). Dalmau Duch et al., reported a woman with severe reactions despite VIT, whose course required reassessment of culprit species and dosing, showing how recurrent field stings can reveal gaps in species identification or VIT protocol and prompt re-tailoring (160). In patients with clonal mast-cell disorders, Jarkvist et al., noted that severe field-sting reactions combined with elevated baseline tryptase frequently triggered VIT intensification or prolonged therapy, again using sting events as real-world indicators of residual risk (116).

Studies using sting challenge to assess VIT efficacy in patients with mastocytosis or elevated BTC indicate that VIT reduces systemic reactions but is less effective than in the general venom-allergic population. Niedoszytko et al., reported systemic reactions in 23.9% sting challenges and a cumulative reaction rate (sting challenges plus field stings) of 28%, corresponding to an estimated protection rate of ∼72%∼ (121) Rueff et al., found that BTC >20 µg/L and/or mastocytosis in the skin (MIS) was a significant, though not the strongest, predictor of VIT failure; however, in patients without features of systemic mastocytosis an isolated elevated tryptase did not increase the risk of VIT failure (122).

Larger analyses help define when sting challenge is most informative. A multicentre retrospective study of patients who had completed VIT found that controlled sting provocation confirmed long-term protection in the majority and identified a small subgroup who remained susceptible, particularly those with very severe initial reactions or mastocytosis. The authors concluded that sting challenge should be reserved for selected high-risk or occupationally exposed patients in whom the result would significantly influence management decisions, rather than used routinely (123). Prospective maintenance-VIT studies reached similar conclusions, regarding sting challenge as the gold standard for verifying efficacy but recommending its use only when the result will change therapy (e.g., continuation, intensification or discontinuation of VIT) (119).

Technical work has addressed standardization of sting delivery. Feás et al., developed “StingReady,” a 3D-printed device that immobilizes live insects and allows controlled sting application under standardized conditions. In a pilot study, the system proved feasible, safe and acceptable, enabling reproducible positioning and timing and facilitating insect transport and short-term maintenance for in-hospital use (124). In a cohort of 42 hornet-allergic patients, Bertlich et al., used sting provocation to compare protection at 100 µg vs. 200 µg maintenance doses and showed that some high-risk patients required higher doses to achieve tolerance, illustrating how sting challenges can guide individualized dose escalation (19).

Several reviews have synthesized the evidence and refined indications. Jakob et al., emphasized that while sting challenges have greatly advanced understanding of VIT efficacy and natural history, the risk of systemic reactions, including rare life-threatening events, means that diagnostic benefit must clearly outweigh potential harm. Less invasive investigations, history, skin tests, sIgE, CRD and BAT, should be exhausted first, and sting challenge should be reserved for cases in which management decisions (e.g., VIT discontinuation, dose change, clearance for high-risk occupations) depend on definitive *in vivo* results (26). Ruiz-León et al., focused specifically on controlled sting challenge and underscored its role in double-sensitized patients, where culprit identification is difficult using *in vitro* and skin tests alone, but stressed the need for stepwise, risk-adapted algorithms (75).

Aßmus provided detailed practical recommendations, noting that sting provocation is generally confined to specialized allergy centres, performed only with clear indication, continuous monitoring and immediate resuscitation capability. The review emphasized that even after a negative sting challenge, patients with previous severe reactions or mast-cell disorders should continue to carry emergency medication and, in some cases, remain on VIT for several months, since protection is high but not absolute (125).

Altogether, live-insect provocation and controlled sting challenge are highly informative but high-risk tools in Hymenoptera venom allergy. They provide the most direct proof of clinical reactivity or protection and can refine decisions on VIT initiation, continuation, dose adjustment and discontinuation, especially in complex or high-stakes situations such as severe initial reactions, occupational exposure or underlying clonal mast-cell disease.

### Emerging and experimental biomarkers in Hymenoptera venom allergy

Osteopontin (OPN) has been proposed as a functional biomarker of successful VIT and a potential regulator of IgE-mediated responses. In a microarray study of PBMCs from untreated patients, early VIT (≤1 year) and long-term post-VIT (5–6 years), Konno et al., identified OPN among 1,751 differentially expressed genes, with stepwise upregulation (1.6-fold in early VIT, 7-fold in completed VIT) confirmed by PCR; monocytes were the main OPN producers, and several OPN-related signalling genes (CD44, integrin α4, IFN-γR, IL-1R, TNFR2, GM-CSF receptor, urokinase) were co-upregulated (126). Serum OPN was significantly higher after completed VIT, and in eight patients followed for 24 months OPN rose progressively, inversely paralleling venom-specific IgE. Given OPN's role as a Th1-associated cytokine and its genetic links to IgE regulation, the authors suggested OPN as a candidate long-term VIT biomarker, while emphasizing the need for larger functional and clinical studies (126).

Arachidonic-acid metabolites, particularly prostaglandin D_2_ (PGD_2_) derivatives, have been explored as indicators of mast-cell activation. In 19 venom-allergic children undergoing rush VIT, Cichocka-Jarosz et al., found that baseline serum tryptase, but not PGD_2_ metabolites, predicted systemic reactions; PGD_2_ derivatives showed limited predictive performance and paradoxically lower urinary levels in reactors, limiting their usefulness for monitoring VIT risk (127). In a subsequent case-control study, venom-allergic children had higher plasma 9α,11β-PGF_2_ and lower urinary levels than controls, with ROC AUCs ≈0.80–0.81, and urinary PGD-M and 9α,11β-PGF_2_ decreased with higher Mueller grades (3). This pattern of elevated plasma but reduced urinary PGD_2_ metabolites appears characteristic of pediatric venom allergy, although practical diagnostic implications remain uncertain (3, 127).

Immunoregulatory markers such as soluble CTLA-4 (sCTLA-4) and IL-10 have also been evaluated. Riccio et al., followed 94 venom-allergic patients across conventional, rush and ultra-rush VIT and observed that sCTLA-4, detectable in all patients at baseline, fell sharply and remained low 12 months after induction, whereas IL-10 rose significantly immediately after induction but returned toward baseline within one year. These kinetics suggest that sCTLA-4 may mark sustained T-cell regulation, while IL-10 reflects early regulatory activation independent of protocol (128). Ruiz-León et al., found that tolerant beekeepers, stung ≥50 times/year without relevant reactions, had expanded Helios-negative CD4+CD25^high^CD127^low^ Tregs, increased CTLA-4+ Tregs, reduced Th1/Th2/Th17 subsets and higher IL-10 production, defining an immunoregulatory phenotype associated with natural tolerance (75). Together, these findings support sCTLA-4, IL-10 and Helios-negative/CTLA-4+ Tregs as promising markers of tolerance induction and maintenance (75, 128).

CD30 and its soluble form sCD30, reflecting Th2-type activation, have also been studied. In 14 venom-allergic patients and 61 controls, Foschi et al., found markedly elevated baseline sCD30 in allergic patients compared with controls, followed by rapid decline during VIT, with levels approximating controls by month 3. sCD30 did not correlate with specific IgE, reaction severity or venom type but was proposed as a convenient marker of VIT-induced immune deviation (129). In a multiplex analysis of 37 inflammatory proteins, Packi et al., showed that sCD30/TNFRSF8 and sTNF-R1 were significantly lower in venom-allergic patients than in sIgE-negative controls; although individual AUCs were modest (0.65–0.69), CD30-related markers may contribute to multiplex diagnostic panels (130).

Highly sensitive detection of the somatic KIT p.D816V variant in peripheral blood has transformed risk stratification by revealing otherwise occult clonal mast-cell disorders. In 839 venom-allergic patients, Luzar et al., detected KIT p.D816V in 15%, including ∼70% with normal BST (≤11.4 ng/mL), demonstrating that tryptase alone is insufficient to detect clonal disease (131). KIT-positive patients had more severe reactions (73% Mueller grade IV vs. 51% in KIT-negative) and higher VIT failure rates; 35% of treatment failures vs. 16% of successes were KIT-positive, and 80% of KIT-positive failures had allele burdens >0.01% (131). In a multicentre cohort of 1,319 patients, Korošec et al., found KIT p.D816V in 21.6% and hereditary α-tryptasemia (HαT) in 7.6%; 27% carried KIT and/or HαT, and 72.6% of KIT-positive patients presented with Ring–Messmer grade III–IV reactions compared with 39.1% of KIT-negative. Most KIT-positive patients had BST <11.4 ng/mL, yet all 41 who underwent bone marrow evaluation had clonal mast-cell disease; combined KIT p.D816V plus HαT conferred the highest risk (87.1% with grade III–IV reactions) (132). Šelb et al., found KIT p.D816V in 8% of venom-allergic patients with normal BST and showed that in this group KIT positivity and age, but not BST or HαT, independently predicted Mueller grade IV reactions, with KIT-positive patients more often experiencing loss of consciousness and fewer skin symptoms (133). Fatal case series confirm the clinical importance: in three patients with fatal sting anaphylaxis, KIT p.D816V-positive clonal mast-cell disease was identified post-mortem, and epidemiologic data indicated that most pathology-confirmed fatal cases harboured KIT p.D816V despite often normal tryptase (134). Collectively, these studies support routine sensitive KIT p.D816V testing, combined with HαT genotyping, in patients with moderate-to-severe venom allergy to unmask clonal mast-cell disease, refine risk and guide decisions on lifelong VIT and intensified emergency management (131–134).

Immunophenotyping of lymphocyte subsets and allergen-reactive T-cell populations provide additional mechanistic and potential diagnostic insight. In a classic longitudinal study of 10 venom-allergic patients undergoing rush hyposensitisation, Tilmant et al., found early transient shifts in CD4+ subsets from CD4+CDw29+ helper/inducer toward CD4+CD45R+ suppressor/inducer cells, along with decreased CD25 and CD23 and parallel changes in CD8+ subsets, suggesting early induction of a more regulatory T-cell phenotype (135). Ruiz-León et al., showed that tolerant beekeepers have expanded Helios-negative and CTLA-4+ Tregs with reduced Th1/Th2/Th17 populations and increased IL-10, emphasizing inducible Tregs in natural tolerance (75). Kearns et al., demonstrated that allergen-reactive γδ T cells exhibit stimulus-specific transcriptional programmes and repertoire patterns, with allergic donors showing enriched IFNG+ Vδ2 populations in response to cockroach antigen, indicating that unconventional T-cell subsets may contribute to allergic inflammation (136). Subramaniam et al., identified CD1a-restricted, lipid-specific T cells that respond to bee and wasp venoms and to venom phospholipase A_2_ with IFN-γ, GM-CSF and IL-13 secretion; these cells increase in frequency and cytokine production during early VIT and drop back to baseline once tolerance is established, suggesting a dynamic effector population that could be monitored (137). In mastocytosis cohorts, Górska et al., reported that reduced expression of B3GAT1 (encoding the CD57/HNK-1 epitope) correlated with a history of insect-venom anaphylaxis, pointing to lymphocyte glycosylation patterns as additional exploratory risk markers (138).

High-dimensional molecular and proteomic profiling is being pursued to identify robust biomarkers of disease status and VIT response. Karpinski et al., used whole-blood microRNA microarrays (2,549 miRNAs) in 13 patients before and 12 months after ultrarush VIT and in 14 controls; global miRNA patterns were highly stable, and after multiple-testing correction no miRNA remained significantly deregulated, with two candidates failing validation, suggesting that whole-blood miRNA signatures may be insufficiently sensitive to capture VIT-induced changes (139). In contrast, Demšar Luzar et al., applied RNA sequencing to whole-blood transcriptomes of 19 patients undergoing honeybee ultra-rush VIT and observed broad down-regulation of inflammatory and Th2/Th17-related genes in successfully treated patients, whose post-therapy profiles resembled healthy controls more than treatment failures. They identified C4BPA, consistently overexpressed 10–14-fold in successful VIT vs. controls and failures, and a downregulated chimeric transcript RPS10–NUDT3 as potential blood markers of long-term tolerance, whereas previously proposed markers such as OPN, CD30, IL-4 and IFN-γ did not show significant changes in this cohort (140).

Proteomic MALDI-TOF MS profiling has also revealed treatment-associated signatures. In a longitudinal VIT cohort, Matysiak et al., documented dynamic changes in several proteins, including fibrinogen α chain, complement C4-A, complement C3, filamin-B, kininogen-1, myosin-9 and ITIH1, with the most pronounced alterations in high-IgG4 responders, suggesting that VIT modulates coagulation–complement–acute-phase pathways (141). A separate case–control proteomic study by Matuszewska et al., identified fibrinogen α chain, factor XIII A chain, complement C4-A and ITIH4 as discriminative proteins between venom-allergic patients and controls, again implicating coagulation and complement proteins in systemic inflammatory signatures of venom allergy (142).

Additional mechanistic work outside human venom allergy, such as the insect-bite hypersensitivity model in horses by Jebbawi et al., shows that combined blood-leukocyte patterns and lesional skin cytokine/chemokine signatures (e.g., IL-4, IL-5, IL-13, IL-31, IL-10, TSLP, histamine receptors) can provide strong diagnostic performance (AUC >0.8 for several targets), supporting the feasibility of integrated systemic–tissue profiling approaches that could ultimately be translated to Hymenoptera venom allergy (143). Finally, Korošec et al., reported that acute-phase molecular markers such as CCL2, FCER1A and CPA3 mRNA, together with absolute basophil counts, discriminated venom-induced anaphylaxis from convalescent and healthy states with very high accuracy (AUC 0.99–0.93), outperforming tryptase and BAT, and thus highlight the potential of combined chemokine–transcriptomic signatures for acute diagnostics (98).

Overall, despite the increasing number of proposed biomarkers in Hymenoptera venom allergy and venom immunotherapy, most remain exploratory and lack sufficient validation for routine clinical use. Most findings come from small, diverse cohorts or single-center studies and have not been replicated in independent groups. Importantly, for many candidates, including OPN, PGD_2_ metabolites, immunoregulatory markers (such as sCTLA-4, IL-10, Tregs), CD30, and high-dimensional molecular signatures, standardized tests, validated cut-off points, and meaningful clinical thresholds are not yet established. As a result, their ability to predict key outcomes, like the severity of allergic reactions, response to VIT, long-term protection, or relapse after stopping therapy, remains uncertain or unproven. Additionally, some markers are influenced by broader inflammatory or immune processes, limiting their specificity for venom allergy. Currently, only a few markers, such as KIT p.D816V mutation and basal tryptase, show consistent validation and relevant prognostic value, especially for identifying patients at higher risk of severe reactions. Overall, the existing evidence suggests that most emerging biomarkers are mainly useful in research at this point, highlighting the need for large, prospective, multicenter studies to determine their diagnostic and prognostic usefulness and to facilitate their integration into standardized clinical protocols.

### Quality of life questionnaires in patients with Hymenoptera venom allergy

Currently the most validated tool for assessing quality of life in patients with HVA is the Vespid Allergy Quality of Life Questionnaire (VQLQ) developed by Elberink et al., (144). This questionnaire has been widely acnowledged and translated to many languages (21, 145–150), however it is not adapted for patients with bee venom allergy as there are differences in the risk factors of beeing stung between those groups. Authors suggested that this test may be still useful in patiens allergic to bee venom who are not beekeepers (144). Interestingly, this questonarie has been also validated for other Hymenoptera species such as Jack jumper ants.

Elberink et al., using this questionnaire, demonstrated that patients treated with VIT experienced a significant improvement in their QoL compared with the other group, which was given an EpiPen and did not undergo immunotherapy. Moreover, prescribing an EpiPen to patients who chose not to receive VIT was associated with a deterioration in QoL. Interestingly, these findings were not influenced by the severity of the initial reaction or by the occurrence of subsequent stings (144).

Moreover, Eitel et al., showed how the sting challenge influences the QoL in patients with HVA. In their study patients who tolerated a sting challenge had the best score in VQLQ and patients during VIT who had not yet undergone provocation had better QoL than patients without any treatment. However, this has been only found significant in patients with vespid allergy, as patients allergic to bee venom (especially beekeepers) tend to be less anxious around their insects. This data confirms benefits from VIT and its impact on QoL and further emphasizes additional benefits from performing a sting challenge.

Studies suggest that this questionnaires should be implemented at the start of venom immunotherapy, especially in patients with grade I systemic reaction to sting, who may not qualify for VIT, but due to low QoL may benefit from immunotherapy (Rueff 2023 S2k). Similarly, high score on VQLQ (meaning low QoL) at the end of VIT may prompt us to prolong therapy duration for patient comfort (29).

## Discussion

The diagnostic landscape of Hymenoptera venom allergy (HVA) has advanced substantially, yet important challenges persist. Traditional elements, patient history, skin testing and serum IgE, remain fundamental, but each has clear limitations. Histories are often imprecise, with frequent misidentification of the culprit insect, and high-exposure groups such as beekeepers may remain unevaluated despite recurrent systemic reactions. These shortcomings emphasize the need for structured diagnostic pathways that integrate detailed clinical context with validated laboratory tools.

Skin prick and intradermal tests remain central because of their high sensitivity, but specificity is limited by cross-reactivity, especially among vespid venoms. Extract-based tests do not reliably distinguish overlapping sensitization patterns and may perform poorly soon after a sting or in patients with mast-cell disorders. Variability in extract composition, including underrepresentation of Api m 10 and other key allergens, further complicates interpretation. Consequently, negative or equivocal skin tests do not reliably exclude clinically relevant allergy, whereas positive results may reflect asymptomatic sensitization.

Serum IgE and immunoenzymatic assays complement skin tests but share similar constraints. Quantitative IgE correlates poorly with clinical severity: high titers may persist long after clinical tolerance is established, whereas low levels do not rule out severe reactions, particularly in patients with mast-cell disorders or in those reacting during VIT despite stable serology. CCDs frequently generate misleading double-positive results. Component-resolved diagnostics improve species attribution, but ambiguous patterns still occur in highly exposed individuals, underlining that IgE must always be interpreted within clinical context.

IgG4 responses, long viewed as markers of tolerance and progress during immunotherapy, have proven inconsistent as predictors of protection. Although IgG4 typically rises during successful VIT and often parallels reductions in IgE and skin reactivity, neither its magnitude nor trajectory reliably reflects sting outcomes or long-term protection. Overall, humoral markers, whether IgE or IgG4, cannot serve as stand-alone surrogates of functional immune protection.

Among established biomarkers, tryptase, acute and basal, remains one of the most informative. Elevated basal tryptase correlates with more severe sting reactions and can indicate underlying clonal mast-cell disease or hereditary α-tryptasemia, although elevations may also arise from non-clonal conditions. Despite its value as a severity risk marker, tryptase does not consistently predict adverse events during VIT. Acute tryptase measurements retain diagnostic importance in anaphylaxis, and updated interpretive criteria (20% + 2 rule) enhance sensitivity, but tryptase alone is insufficient for comprehensive risk stratification.

Emerging biomarkers offer additional perspectives. Osteopontin may mark immunologic modulation during VIT; PGD_2_ metabolite patterns provide insight into mast-cell activity; and regulatory markers such as sCTLA-4, IL-10 and specific T-reg subsets illuminate mechanisms of natural and therapy-induced tolerance. CD30-related molecules, although variable across cohorts, appear to reflect Th2 activity and VIT-induced immune deviation. Highly sensitive detection of clonal mast-cell markers such as KIT p.D816V and identification of hereditary α-tryptasemia have markedly improved recognition of underlying mast-cell disease and refined risk prediction. Broader transcriptomic, proteomic and immunophenotypic profiling has revealed treatment-associated molecular signatures and potential candidate markers, but reproducibility and standardization remain limited, and these methods are still largely investigational.

Sting-challenge testing, whether natural or controlled, continues to provide the most direct assessment of true clinical reactivity and VIT efficacy. Ethical, safety and logistical constraints limit its use, but when carefully indicated and performed in specialized centres, sting provocation can identify patients who remain unprotected, guide dose adjustments or prolongation of VIT and support decisions in high-stakes contexts such as occupational exposure or clonal mast-cell disease. New technologies intended to standardize sting delivery, such as StingReady, may improve safety and reproducibility, though their broader adoption is uncertain.

Across all modalities, a consistent theme emerges: no single test provides adequate diagnostic certainty in isolation. Clinical history remains indispensable; venom-specific IgE and skin tests provide foundational sensitivity; component-resolved diagnostics refine species identification and help interpret complex sensitization patterns; tryptase and clonal markers inform mast-cell–related risk; functional assays such as BAT and MAT document effector-cell responsiveness; and sting challenges deliver definitive evidence of protection or residual susceptibility. Emerging biomarkers and molecular signatures may add further layers of information but currently require validation before routine implementation.

Looking forward, major unmet needs include biomarkers that can reliably predict treatment safety and durable protection, tools that better distinguish clinically relevant from irrelevant sensitization and standardized algorithms that integrate molecular, cellular and clinical parameters. Advances in mast-cell biology, immunophenotyping, transcriptomics, proteomics and functional diagnostics, together with refined clinical algorithms, may ultimately support a more precise, personalized and predictive diagnostic framework for HVA, one capable not only of confirming allergy but also of stratifying risk, guiding therapy and monitoring immunologic change over time.

Despite significant progress, the diagnostic and biomarker landscape in Hymenoptera venom allergy is still limited by several key factors. Variability in methods, such as differences in allergen extracts, assay platforms, and interpretation criteria, further hampers comparability and standardization. Most emerging biomarkers lack external validation, and clinically relevant cut-off values have yet to be determined. Importantly, their ability to predict critical outcomes, like reaction severity, treatment safety, long-term protection, or relapse after venom immunotherapy, remains inconsistent or unproven. Additionally, many markers indicate nonspecific immune activation, reducing their specificity and increasing the risk of confounding. Practical challenges, including limited availability, high costs, and the need for specialized expertise, also restrict clinical use. As a result, current diagnostics rely on combining multiple imperfect tools rather than on robust, standalone biomarkers.

### Limitations of the study

The findings of this review should be understood considering several limitations. Many of the included studies are retrospective and observational, which are susceptible to selection bias and confounding, thereby limiting causal inference and generalizability. Furthermore, variability in study design and populations, along with possible publication bias, may affect the overall results. Additional prospective and randomized studies are necessary to reinforce the evidence base.
